# Post-zygotic selection against parental genotypes during larval development maintains all-hybrid populations of the frog *Pelophylax esculentus*

**DOI:** 10.1186/s12862-015-0404-3

**Published:** 2015-07-04

**Authors:** Heinz-Ulrich Reyer, Christian Arioli-Jakob, Martina Arioli

**Affiliations:** Institute of Evolutionary Biology and Environmental Studies, University of Zürich, Winterthurerstrasse 190, Zürich, CH-8057 Switzerland

**Keywords:** Assortative mating, Environmental conditions, Food supply, Fertility, Genetic diversity, Larval development, Pre- and post-zygotic selection, Survival

## Abstract

**Background:**

Hybridization between two species usually leads to inviable or infertile offspring, due to endogenous or exogenous selection pressures. Yet, hybrid taxa are found in several plant and animal genera, and some of these hybrid taxa are ecologically and evolutionarily very successful. One example of such a successful hybrid is the water frog, *Pelophylax esculentus* which originated from matings between the two species *P. ridibundus* (genotype RR) and *P. lessonae* (LL). At the northern border of the distribution all-hybrid populations consisting of diploid (LR) and one or two triploid (LLR, LRR) frog types have been established. Here, the hybrid has achieved reproductive independence from its sexual ancestors and forms a self-sustaining evolutionary unit. Based on the gamete production of these hybrids, certain mating combinations should lead to LL and RR offspring, but these parental forms are absent among the adults.

**Results:**

In order to investigate the mechanisms that maintain such an all-hybrid system, we performed a field study and a crossing experiment. In the field we sampled several ponds for water frog larvae at different developmental stages. Genotype compositions were then analysed and life-history differences between the genotypes examined. In the experiment we crossed diploid and triploid males and females from different ponds and determined fertilization success as well as development speed and survival rates of the offspring under high, medium and low food availability. In both parts of the study, we found numerous LL and RR offspring during the egg and early larval stages; but the frequency of these parental genotypes decreased drastically during later stages. In natural ponds almost all of them had disappeared already before metamorphosis; under the more benign experimental conditions the last ones died as juveniles during the following year.

**Conclusions:**

From the combined results we conclude that the absence of parental genotypes in all-hybrid populations is due to post-zygotic selection against them, rather than to pre-zygotic mechanisms that might prevent their formation in the first place. For this post-zygotic selection, genetic mechanisms resulting from low genetic diversity and fixation of deleterious mutations seem to be a more likely explanation than ecological factors.

## Background

Hybridization, the production of offspring between individuals of different species, is quite common in nature (reviewed by [1, 2]), and the number of recognized taxa that are of hybrid origin is increasing, mostly due to the improvement of molecular techniques [3]. Yet, the evolutionary importance of hybridization has been strongly debated among biologists for many decades. Whereas botanists have long accepted hybridization as an important evolutionary force in speciation [4], zoologists traditionally neglected such an influence and considered it an evolutionary dead end [5]. Animal hybrids are usually thought to have lower fitness than the parental species due to endogenous (genetically based) or exogenous (environmentally based) selection acting upon them. Endogenous selection is based on the concurrence of two different genomes, which often results in developmental instability, sterility or even death of the newly formed hybrid [6, 7]. Exogenous selection against hybrids can result from the fact that their morphological, physiological and/or behavioural traits are often intermediate between those of their two parental species [8–11]. This will leave them at a disadvantage in the parental habitats; successful establishment may only be possible where intermediate habitat conditions exist [12].

However, hybrid fitness in relation to fitness of the parental forms can vary greatly, and cases of hybrid superiority to at least one parental form have been well documented (reviewed in [13]). Such hybrid superiority is often caused by heterosis effects and therefore restricted to (or at least highest in) the F1 generation [14]. As a result, the hybrid taxon will only persist under conditions of repeated primary hybridization. To be of evolutionary importance the hybrid either has to backcross with at least one of the parental species and introduce new genes into the parental gene pool; or the hybrid has to become an evolutionary significant unit (ESU) of its own by either outcompeting the parental species and overtaking their niches or by developing adaptations to a new specific niche and, as a result, restrict gene flow from other lineages within the higher organizational level of the species [15]. Thanks to increased research in this field and improved genetic tools, meanwhile several hybrid taxa have been identified that are ecologically very successful and seem to have reached an evolutionarily old age [16–18].

### The palaearctic water frog complex (*Pelophylax*)

One of the systems where the hybrid taxon has existed over a long time period, i.e., 10,000-50,000 years [19], and is ecologically successful (in the sense that it is geographically widespread) is the European water frog *Pelophylax esculentus*, which was formed through repeated hybridization between two parental species *P. lessonae* (genotype LL) and *P. ridibundus* (genotype RR). The hybrid nature of *P. esculentus* was first shown by Berger [20, 21] through biometric analyses and breeding experiments; further investigations revealed that its usual reproductive mode is hybridogenetic [22]. Hybridogenesis is characterized by premeiotic exclusion of one parental genome during gametogenesis, followed by endomitosis of the remaining genome (i.e., its duplication prior to cell division) and its clonal transmission to gametes that all carry identical chromosomes [23, 24]. Offspring from matings between hybrids usually do not survive, due to the accumulation of mutations on the clonally inherited genomes, which can impair development through homozigosity of recessive deleterious alleles at particular loci and/or general deterioration from high mutational load, independent of homozigosity [19, 25, 26]. Therefore, production of viable hybrid offspring requires that *P. esculentus* acts as a sexual parasite that restores hybridity by mating with the respective parental species (the sexual host) whose genome was excluded. Thus, *P. esculentus* represent ‘hemiclones’ [27] in which selection against deleterious mutations is weak because the clonal genome transmitted by the parasite is constantly sheltered by the sexual genome of the host [26].

Whether or not genome exclusion is induced and, if so, which parental genome is excluded and to what extent may vary with population composition, ploidy and geographic area [28, 29]. Where the L-genome is eliminated and the R-genome transmitted, *P. esculentus* occur in sympatry and mate with the parental species *P. lessonae* (LE-system); where the reverse pattern exists (elimination of R, clonal transmission of L), hybrids co-occur and mate with *P. ridibundus* (RE-system) [30]. In both these systems, the vast majority of the hybrids are diploid (genotype LR); and if triploids are present at all, they occur in proportions of less than 15 % (for details see Appendix 1 [31]). Models have shown that stability of these mixed hybrid-parental systems is very sensitive to several factors, such as mating preference, female fecundity and larval performance of the involved taxa [32–34].

**Fig. 1:**
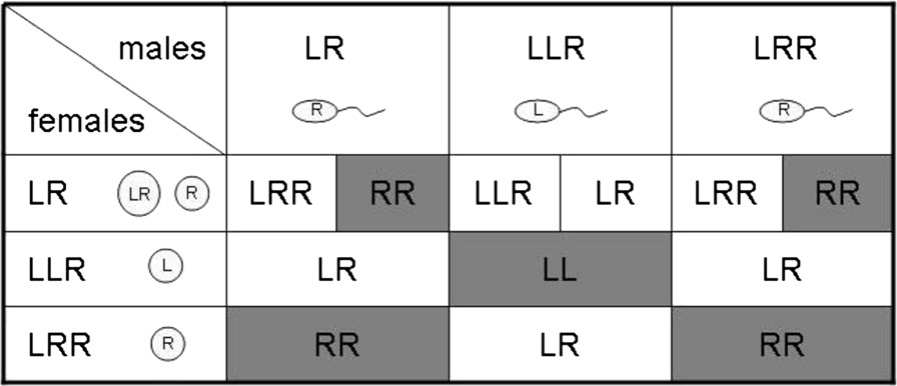
Typical gamete production in female and male genotypes from all hybrid *P. esculentus* populations and offspring types arising from the nine potential mating combinations. Female LR can produce both diploid eggs and haploid eggs. Genotypes in grey boxes do not occur among the adults in the population although they are initially produced

A third population type (EE-system) is represented by all-hybrid populations of *P. esculentus*. They dominate in the northern part of the distribution area, including our study area in southern Sweden; but they are also found in some areas of Central and Eastern Europe (reviewed by [35, 36]). These populations consist of diploid hybrids (genotype LR), plus one or two triploid forms (LLR and/or LRR); tetraploid LLRR hybrids are very rare exceptions that amounted to only 0.3 % of all sampled frogs [37]. While the formation of diploid and triploid hybrids is well understood [38–41], the absence of parental genotypes from these all-hybrid systems remains a puzzle. Based on the typical gamete production patterns in these populations, both LL and RR offspring are to be expected (Fig. 1); but despite of extensive sampling of 3165 frogs we have not found adults with these genotypes.

In this study, we tested the following two hypotheses for the absence of *P. lessonae* and *P. ridibundus* in all-hybrid *P. esculentus* populations.

1. Offspring with parental genotypes are not produced from the very beginning, because of assortative mating and/or unsuccessful fertilization of eggs (pre-zygotic selection).
2. All genotypes are initially produced, but those of the parental species do not survive to the adult stage (post-zygotic selection).

To distinguish between these two hypotheses we combined results from a field study, where we sampled 12 ponds for water frog offspring at different developmental stages, with results from an experiment, where we raised tadpoles emerging from artificial crosses between adult frogs of known genotypes. In both parts of the study, we analyzed the genotype composition at different developmental stages to test whether the relative frequencies of the genotypes changed from eggs through tadpoles and metamorphs to 1-year old juveniles. In the experimental part, we further identified the produced gametes by comparing the genotypes of parents and offspring and recorded genotype- and sex-specific fertilization rates. We also raised the tadpoles under three feeding regimes with high, medium and low food availability, respectively, and tested for a genotype x environment effect to see whether genotype-specific development varies with environmental conditions.

Ours is not the first study to document changes in larval genotype proportions in all-hybrid populations (e.g., [42]), but it is the first one to systematically monitor such changes from the egg to the adult stage under natural and experimental conditions for all five genotypes that theoretically could be produced in such populations (LL, LLR, LR, LRR and RR). Moreover, with the exception of one study that used microsatellite markers [43], earlier studies on larval genotypes were based on measures that cannot reliably distinguish between genotypes, either because the distributions of the respective parameters overlap as for egg size, morphometric indices and erythrocyte size or the resolution is not fine enough as in allozyme analyses [44–46]. By combining microsatellite and flow cytometry analysis we were able to overcome these limitations and achieve unambiguous genotype identification (even of rare forms) at various developmental stages.

## Results

### Survival and development in natural ponds

**Fig. 2:**
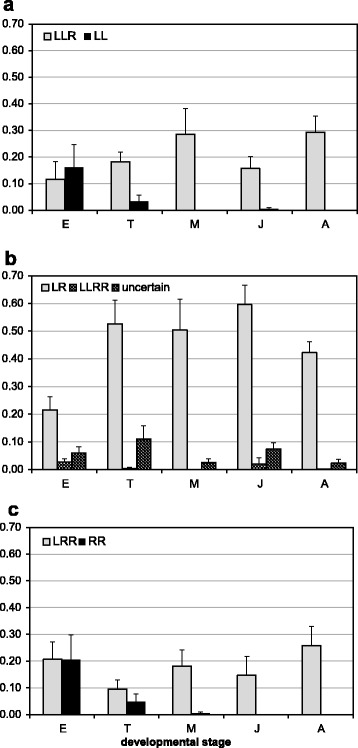
Average proportions (± SE) of different genotypes in natural ponds at four developmental stages: E = eggs, T = tadpoles, M = metamorphs, J = 1-year old juveniles A = adults. Grey bars represent the three hybrid types that are surviving and dominate among adults (LLR in **a**), LR in **b**) and LRR in **c**); the black bars show values for the parental species LL (**a**) and RR (**c**) that are gradually disappearing. In **b**) the stippled bars represent LLRR hybrids that are found among adults in low proportions, the hatched bars denote proportions of uncertain genotypes, i.e., those that could not unambiguously be identified in the microsatellite analysis (cf. Methods)

At the fertilized egg stage, we found all offspring types that are to be expected from the gamete type combinations shown in Fig. 1. Only about half of the sample was comprised of the three genotypes that characterize all-hybrid populations (LR, LLR and LRR); the other half was made up by types that exist among adults only very rarely (LLRR hybrids and uncertain genotypes) or not at all, such as the parental forms (LL, LLL, RR and RRR) (Fig. 2). After the egg stage, the parental forms disappear almost completely through the tadpole to the metamorph and juvenile stages (Fig. 2a,c). Among metamorphs we found no LL and only one RR, while among juveniles the reverse was true. This decrease in parental genotypes is paralleled by an increase in the proportion of LR hybrids (Fig. 2b). The changes are reflected by significant effects of stage on proportions of LL, RR and LR (Table 1). Pairwise comparisons show that proportions in the egg stage differ significantly from those in the metamorph and juvenile stage for all three genotypes (all *P* ≤ 0.028), and for LR and RR also from those in the tadpole stage (both *P* ≤ 0.024). Proportions did not differ between tadpole, metamorph and juvenile stages for any of the three genotypes. For all other genotypes (LLR, LRR, LLRR and uncertain) proportions were not significantly affected by stage (Table 1); thus, they remained basically constant from eggs to juveniles. Already from the metamorph stage on, the genotype composition was almost identical to that found among juveniles and very similar to the average ratios found among adults in the same 12 study ponds over three consecutive years (2002–2004) (Fig. 2).

**Table 1:** Results from general linear models relating genotype proportions to pond of origin and four developmental stages (eggs, tadpoles, metamorphs and juveniles)

	Stage		Pond	
Genotype	F	p	F	p
LL	5.198	0.006	1.595	0.155
LLR	2.030	0.132	1.537	0.174
LR	6,123	0.002	3.644	0.003
LLRR	2.152	0.116	1.664	0.135
LRR	0.194	0.900	1.641	0.141
RR	8.691	<0.001	1.095	0.400
*(Uncertain*	*2.297*	*0.099*	*1.472*	*0.198)*

Results for the uncertain genotypes are shown in italics and brackets to indicate that its proportions are not independent, because they are the difference between 100 % and the sum of the other six genotypes

Genotype proportions did not differ among ponds, except for LR (Table 1). This difference was mainly due to one pond 102 which had very few LR individuals in all samples. LR proportions in this pond were significantly lower than those in three other ponds (111, 134 and 138; all *P* ≤ 0.023), whereas all other ponds did not differ in pairwise comparisons. However, sample size in pond 102 was very low, so that the difference may be due to stochasticity.

Genotype and pond not only affected survival, but also developmental speed. As shown by Table 2a, the developmental stage of the surviving tadpoles differed significantly among ponds and between genotypes; it was significantly more advanced for hybrids (mean = 34.6, SE = 0.2, *n* = 127) than for parental offspring (mean = 33.2, SE = 0.7, *n* = 13).

**Table 2:** Results from general linear models for a) tadpole, b) metamorph and c) juvenile development in natural ponds

			Developmental stage [97]		Snout-vent length		Body mass	
Stage	Source	df	F	p	F	p	F	p
a) Tadpoles	Genotype	1	4.136	0.044	-	-	-	-
	Pond	9	18.47	<0.001	-	-	-	-
b) Metamorphs	Genotype	2	-	-	7.63	<0.001	6.00	0.003
	Pond	9	-	-	47.74	<0.001	23.98	<0.001
c) Juveniles	Genotype	2	-	-	1.47	0.234	2.46	0.090
	Pond	11	-	-	2.49	0.007	3.64	<0.001

In a) developmental stage (according to [96]) was related to two genotype categories (hybrid and parental offspring) and pond of origin. In b) and c) body mass and size (measured by snout-vent length) were related to three hybrid genotypes (LR, LLR and LRR) and pond of origin

Later in the development, there were hardly any genotypes present, except the three hybrid types (cf. Fig. 2). Among metamorphs, we found overall significant differences regarding snout-vent length and weight (Table 2b). Pairwise comparisons showed that LRR metamorphs were significantly heavier than LR individuals (*P* = 0.002) and larger than both LR and LLR (both *P* ≤ 0.044), whereas other pairwise differences were not significant (all *P* ≥ 0.275). Among juveniles, the genotype effect was not significant (Table 2c), but at both stages size and body mass of individuals differed among ponds.

### Crossing experiment

#### Gamete production

**Fig. 3:**
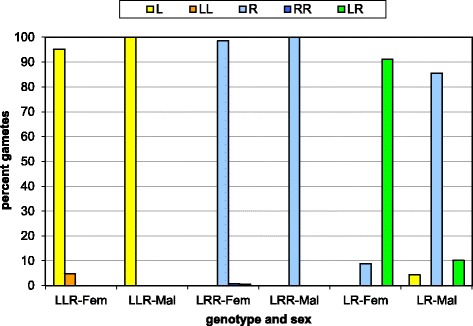
Average proportions of gamete types produced by the crossed male and female hybrids

The gamete types produced by the crossed adults basically confirmed the pattern found in previous studies of all-hybrid populations (Fig. 3): triploid individuals produced haploid gametes with the genome that they carried in two copies (i.e., L in LLR and R in LRR). In males, this was 100 % true for all six LLR and all three LRR and in females for one of four LLR and four of six LRR. The other three LLR females produced a few diploid LL eggs (2.6 – 10.7 %), while one of the six LRR females produced a few diploid RR eggs (4.3 %) and the other one a few LR eggs (3.6 %). In diploid individuals gamete production was more variable (Fig. 3). Four out of 10 females and five out of nine males produced exclusively the gamete types that according to a previous investigation were known to be most frequent in LR hybrids from our study area [40], i.e., LR eggs and R sperm, respectively. The other six LR females produced also haploid R eggs in a frequency ranging from 2.8 to 48.8 %, while the other four LR males showed very diverse gamete ratios. In three of them the expected haploid R sperm amounted to 28.0–89.3 % and the additionally produced diploid LR sperm to 10.7–48.0 %. Two of these males also produced gametes of a third type, namely 10.0–24.0 % haploid L sperm. The fourth unusual LR male had an extremely low fertilization success resulting in only two larvae (one sired by an L and the other by an R sperm), which does not allow a reliable conclusion about the gamete production of this male. Interestingly, all three LR males that produced both haploid L and R sperm came from the same pond (001). As a result of this production of unusual gametes in some males and females, some crosses not only produced the five common and expected offspring types (LR, LLR, LRR, LL and RR); we also found a few rare ones, namely 13 tetraploid LLRR, two LLL and one tadpole each with the genotypes RRR, LLLR and LRRR.

#### Fertilization success and hatching rate

**Fig. 4:**
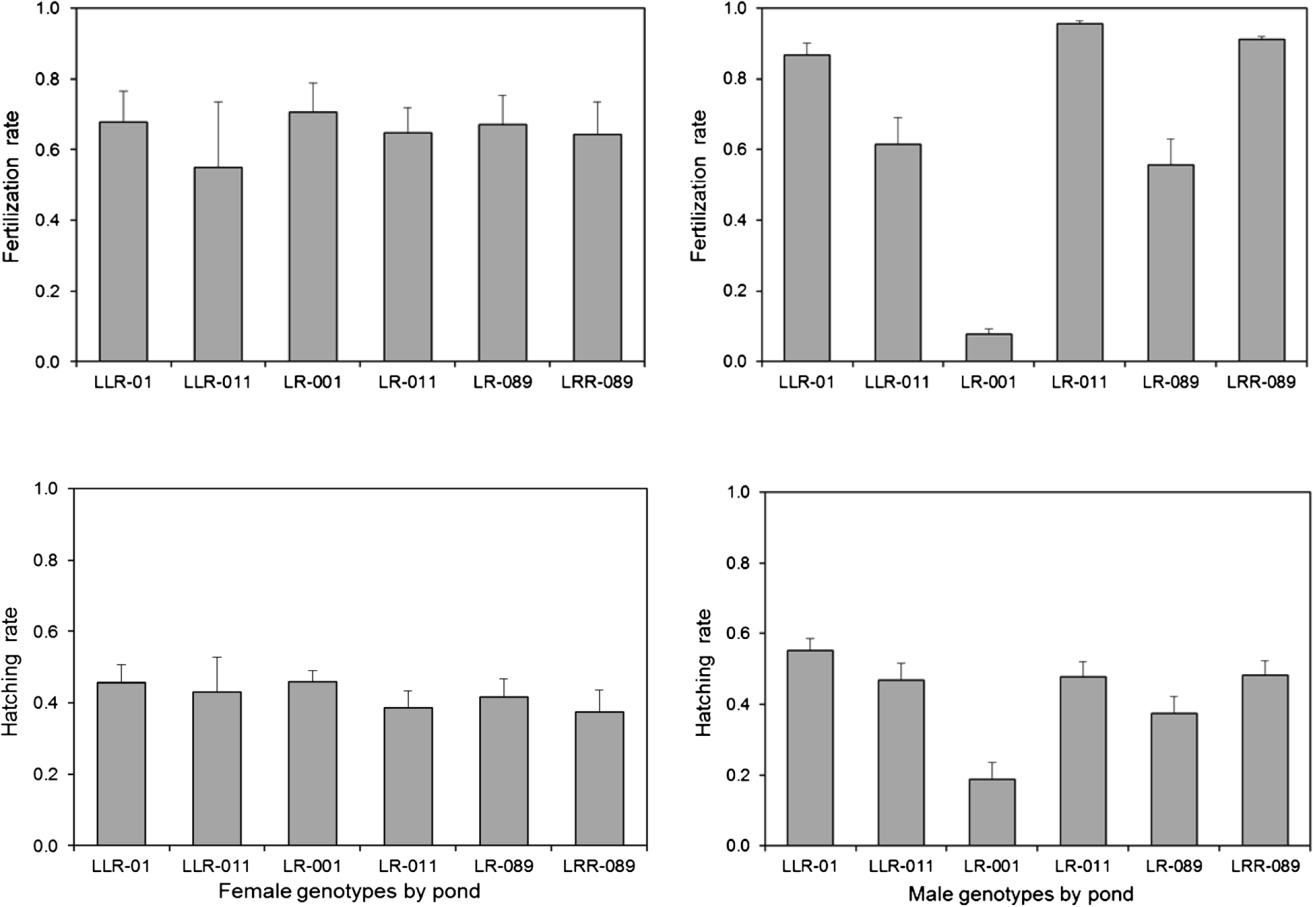
Means in fertilization success and hatching rate for the different genotypes of females and males listed per pond. Error bars represent ±1 SE

Female genotypes did not significantly differ in either fertilization success or hatching rate (Table 3, Fig. 4). On average, 66 % of all eggs were fertilized and 40 % of those developed into hatchlings. Male reproductive success, however, showed clear differences between genotypes and pond of origin. Triploid males of both types (LLR and LRR) had fairly high average fertilization success and hatching rates of their offspring, whereas for diploid LR males the values were significantly lower, with marked differences between the three ponds of origin (Fig. 4). The very low fertilization rate by sperm of LR males from pond 001 (8 %) and the poor hatching rate of their offspring (19 %) translated into only very few surviving tadpoles from crossings involving these males. The interaction between male and female genotype and the crossing type had no significant influence on fertilization success and hatching rate, indicating that initial reproductive success did not depend on which parental genotypes were crossed and whether mothers and fathers came from the same or from different ponds. Since the effect of differing pond origin might be most severe in the homotypic offspring genotypes LL or RR where deleterious mutations in homozygous (same clone) or heterozygous states (different clones) are not countered by the influence of another genome, we also tested for only these two genotypes if crossing type is influencing reproductive success in early life stages; but there were no significant differences in fertilization or hatching rate (*t*_*fertilization*_ = 0.04, *P* = 0.966; *t*_*hatching*_ = 0.33, *P* = 0.741).

**Table 3:** Results from a general linear model for fertilization success and hatching rate in relation to female and male genotype, their interaction and the effect of crossing type, i.e., within versus between pond crossings

		Fertilization success		Hatching rate	
Source of variation	df	*F*	*P*	*F*	*P*
Crossing type	1	0.17	0.681	0.29	0.590
Genotype F (pond F)	4	0.85	0.499	0.94	0.441
Genotype M (pond M)	3	68.17	<0.001	10.37	<0.001
Genotype F x genotype M	4	0.49	0.746	1.94	0.110

#### Survival and development of tadpoles and metamorphs

**Fig. 5:**
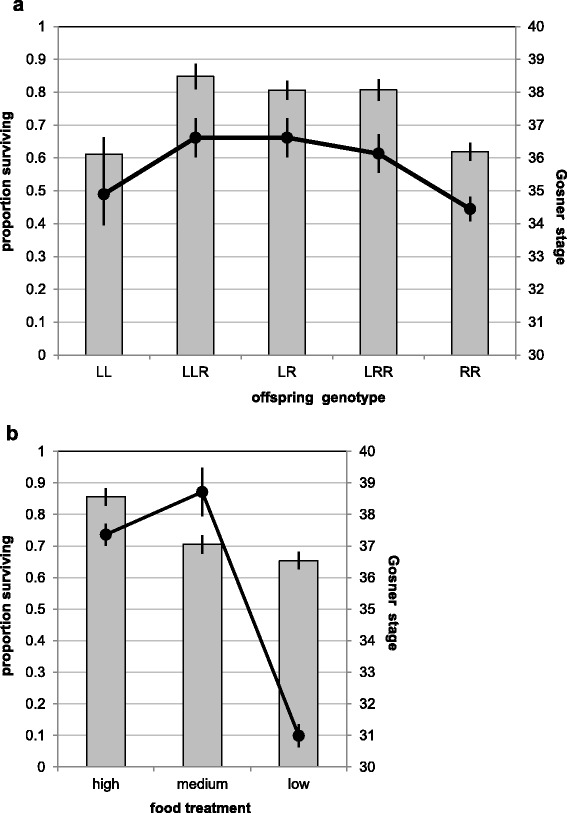
Survival (*bars*) and developmental stage according to [97] until beginning metamorphosis (*black lines*) of (**a**) different offspring types and (**b**) in relation to food treatment. Shown are means ± 1 SE

Due to the production of unusual and multiple gamete types by some males and females (Fig. 3) the progeny from some crosses was composed of different offspring types. Since larval survival was measured per tub, and tubs corresponded to crosses, potential differences in offspring survival and development were analyzed only for those tubs that contained one single genotype. Tadpole survival and development were both significantly affected by offspring type and food treatment (Table 4a, Fig. 5a). Pairwise comparisons showed that both parental genotypes survived significantly worse than the three hybrid genotypes (all *P* < 0.001), whereas there were no differences in comparisons between LL and RR and LLR, LR and LRR, respectively (all *P* = 1.000). Survival increased with the amount of available food (Fig. 5b), being significantly higher in the high food than in the medium and low food treatment (both *P* < 0.001) and a tendency for better survival under medium than under low food (*P* = 0.099). Among those tadpoles that did survive, differences in development paralleled those in survival: tadpoles of the three hybrid types developed faster (indicated by higher Gosner stages) than those of the two parental types (Fig. 5a) and those under better food conditions (high and medium) developed faster than those in the low food treatment (Fig. 5b). For both, survival and development, there was no significant interaction between offspring type and food treatment (although for development there was a tendency); and crossing type also had no effect (Table 4a). This indicates that the response to food conditions does not differ between offspring types and is independent of whether the crossed males and females come from the same or from different ponds.

**Table 4:** Results from general linear models testing for differences in a) tadpole survival and development (according to [97]) until metamorphosis and b) froglet survival and body mass until July of the following year

a) Tadpoles		Survival		Development	
Source	df	F	P	F	P
Offspring type	4	9.81	<0.001	3.51	0.009
Treatment	2	23.10	<0.001	93.36	<0.001
Offspring Type x Treatment	8	1.13	0.121	1.86	0.071
Crossing type	1	2.43	0.346	0.13	0.715
b) Froglets		Survival		Body mass	
Froglet type	1	5.59	0.027	0.66	0.532

In a) independent variables are three hybrid (LLR, LR and LRR) and two parental offspring types (LL and RR), three food treatments (low, medium and high), the offspring x food interaction and the variable “crossing type” describing whether the crossed males and females came from the same or from different ponds. In b) “froglet type” consists of two categories: parental or hybrid offspring

**Fig. 6:**
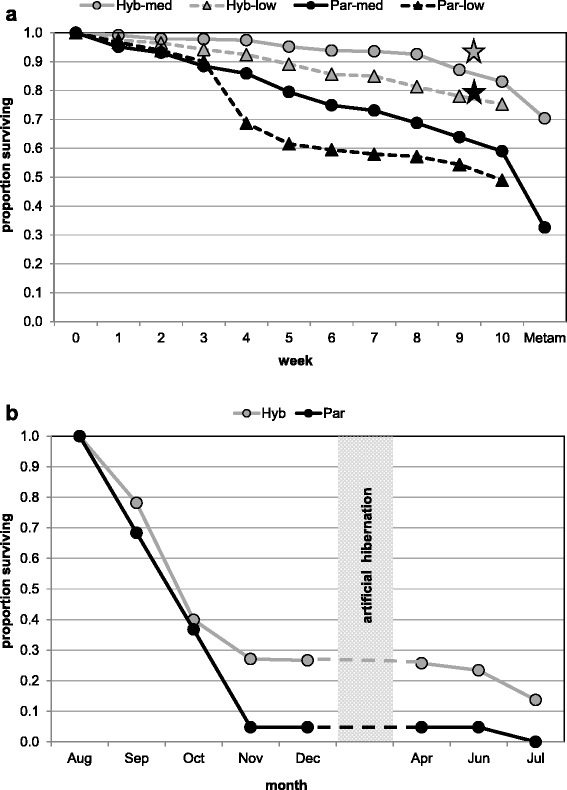
Survival in hybrid (*grey*) and parental (*black*) offspring. In (**a**) the time course of tadpole survival prior to metamorphosis is shown on a weekly basis for medium (*circles*) and low (*triangles*) food treatment. Survival under the high food treatment was measured only once after 9 weeks; the corresponding values for hybrid and parental offspring are indicated by grey and black stars, respectively. In (**b**) only survival of metamorphs emerging from the medium food treatment is shown, because tadpoles from the high and low food treatments were not reared beyond metamorphosis

While the high and low food treatments were terminated at the end of the larval period, tadpoles from the medium food treatment were raised through metamorphosis until July of the following year. During this period, the survival and development patterns found in tadpoles continued through the metamorph and juvenile stages (Fig. 6): at the end of the experiment, parental offspring had survived significantly worse (actually not at all) than hybrid offspring (Table 4b).

## Discussion

Our results from the 12 natural ponds show that initially all offspring genotypes that are possible (based on the existing gamete types) are actually produced. At the fertilized egg stage about 50 % of the sample was comprised of genotypes that existed among adults only very rarely (LLRR hybrids and uncertain genotypes) or not at all, such as the parental forms (LL, LLL, RR and RRR) (cf. Fig. 1); but these genotypes gradually disappeared during subsequent stages. From the metamorph stage on, the genotype composition in all ponds was that of typical EE-systems, i.e., with almost exclusively LR, LLR and LRR hybrids among the adults in ratios that are very similar to those of juveniles and adults (Fig. 2). Therefore, we conclude that the existence and maintenance of all-hybrid populations is due to post-zygotic selection against parental forms (hypothesis 2) during the tadpole stage, rather than to pre-zygotic selection like assortative mating and/or unsuccessful fertilization (hypothesis 1).

### Pre-zygotic selection: assortative mating

The absence of assortative mating in all-hybrid populations of the EE-system is supported by two other studies. In two all-hybrid populations the relative frequencies of the various male x female mating combinations did not differ from those calculated from the genotype proportions under the assumption of random mating [47]. Also, mate choice experiments revealed no preference of diploid and triploid females for males of any of the three hybrid types [48]. These results contrast with those from similar experiments in LE populations: when given a choice between LL and LR males or their calls, LR and LL females both preferred LL males [49–52]. The difference in female choice between the two systems can be explained in both proximate and ultimate ways. In terms of proximate mechanisms, the three hybrid types (LR, LLR, LRR) are more similar to each other in size, coloration and male advertisement calls than diploid hybrids (LR) and the parental species (LL) are, thus making discrimination and choice more difficult [53, 54]. In terms of ultimate reasons, genetic fixation of mate preferences in a particular genotype should be impossible in diploid-triploid all-hybrid populations, because suitable mating partners (i.e., the ones guaranteeing viable progeny types) alternate each generation: diploid LR females producing diploid eggs should choose triploid LLR or LRR males; the resulting triploid daughters should choose diploid LR males etc. [55]. In contrast, in LE-systems, mating with LL males is strongly selected for in LR females, because it is always the only way to produce viable offspring [32, 33].

### Pre-zygotic selection: gamete types and fertilization success

Pre-zygotic selection against certain gamete types and their fusion as a reason for the lack of *P. lessonae* and *P. ridibundus* among adults of all-hybrid populations can also be dismissed. Although, for technical reasons, we had to deduce the produced gamete types from genotyping tadpoles that were 18 days old (see Methods) and, thus, cannot rule out that genotype proportions at this stage differed somewhat from those among the originally produced gametes and zygotes, this potential bias cannot explain the observed population composition. Haploid L and R eggs and sperm, as well as offspring with parental genotypes were produced in large numbers, both in natural ponds (Figs. 2 and 3) and in the crossing experiment (Fig. 5) and with no effect of crossing type; i.e., fertilization success and hatching rate were independent of whether the crossed males and females came from the same or from different ponds. They were also independent of whether diploid LR-females exclusively produced the typical diploid LR- or in addition varying amounts of haploid R-eggs (Table 3). The simultaneous production of LR- and R-eggs by the same diploid LR-female with apparently little fertility impairment has also been described in a number of other *P. esculentus* studies (reviewed by [35, 36]).

This differs from the situation in diploid males where average fertilization and hatching rates were lower than for all other parent types, with values decreasing from males in pond 011 that produced only R sperm through those from pond 089 producing predominantly R- and a small proportion of LR sperm to males from pond 001 with all three gamete types (L, R and LR) in fairly high proportions (Fig. 4). These results strongly suggest that the poor reproductive success in these hybrid males is due to disturbed gametogenesis, as has been already suggested previously [56–59]. In extreme cases, this can lead to the development of abnormal gonads and germ cells [60] and complete male sterility [35, 61].

The link between reproductive success and the extent of gametogenesis problems is further supported by our finding that the high rates of fertilization and hatching in triploid hybrids of both types (LLR and LRR) are paralleled by no aberrant gamete forms in males and only very few in females (Fig. 3). The reason why gametogenesis seems to be more consistent and less disturbed in triploid than in diploid frogs lies in the simpler cytogenetic mechanism of hemiclonal inheritance through so-called “meiotic hybridogenesis” [62, 63]: after the elimination of the unmatched chromosome (“homogenizing elimination”), in triploids *“no compensatory duplication of the remaining genetic material is necessary, as it is in diploids”* [64], and the two homologous chromosomes can go through a normal meiosis.

Although gamete variability of triploids is low among individuals from the same pond, it can differ between populations from different geographical areas, even to the extent that in some populations LLR males typically produce diploid LL instead of haploid L sperm [41, 59, 65, 66]. This and the fact that varying proportions of diploid eggs and sperm have also been found in other studies (e.g., [56, 67]) probably reflects different abilities of the R-genome, the L-genome or both to induce and/or resist genome exclusion (e.g., [68–70]). These differences may result from different primary hybridizations, ecological adaptations and/or reflect variable outcomes of a genomic conflict between segregation distorters (or meiotic driver genes) in the excluding genome and modifier genes in the host genome that counteract meiotic driver genes [71, 72]. Production of diploid gametes may also represent an adaptation of the host genome to prevent exclusion [71]: if, for instance, in an LE-system *P. lessonae* males produced LL rather than L sperm that fertilise R eggs of diploid *P. esculentus*, then genome exclusion would (probably) reverse from L in LR offspring to R in LLR offspring. Although we know of no such LL sperm production in *P. lessonae*, it just may have escaped our attention because of its rareness. This, however, does not exclude the possibility that it can be successful. Production of LL sperm is the usual pattern in LLR males from some populations where it most likely arose from a single event of L genome doubling [73].

In some cases, the outcome of genomic conflicts seems to disturb the normal patterns of gametogenesis in hybrids and, hence, produce unusual eggs and/or sperms. Examples include:

- Resistance to genome exclusion in diploids from some populations [68, 69] and, hence, the possibility for occasional recombination between the L and R genome [56, 74].
- Production of polyploid gametes that upon fusion with haploid ones will lead to unusual offspring types such as the very rare LLL, RRR and the about 7 % uncertain genotypes found in this study or the pentaploid LLLRR reported by [75].
- Progeny with deviations from the parental chromosome proportions expected for diploid (13 + 13) and triploids (13 + 26) (cf. [76]).

It seems plausible to assume that such aberrant offspring encounter developmental problems and are not viable or fertile. But why do the normal parental genotypes (LL and RR) gradually disappear towards sexual maturity? Why is there post-zygotic selection against the parental species in EE-systems, rather than against hybrids as in many other systems? Below, we discuss two potential explanations: environmental factors and genetic mechanisms.

### Post-zygotic selection: environmental factors

Survival and development of *Pelophylax* tadpoles is definitely affected by environmental conditions. This is indicated by various lines of evidence. First, survival rate and developmental stage of tadpoles and the size and body mass of metamophs differed among ponds (Table 2). Second, development improved with improving environmental conditions: it was worst in natural ponds where LL and RR offspring did not even survive to the metamorph stage, whereas under the more benign conditions of the experiments development improved with increasing food availability, and several parental offspring types reached the froglet stage (also found by [77]) and a few even the stage of 1-year old juveniles. Besides food, water temperature has been identified as a factor contributing to developmental differences. Under cold conditions, as they exist at the northern range of the water frog distribution, hybrids not only survive better, they also metamorph faster than the parental species [78, 79] which can have a beneficial influence on survival at later life ages [80].

Based on our study, however, neither food nor temperature can explain the results. The parental genotypes disappeared from all investigated natural ponds, independent of their specific combination of biotic and abiotic conditions (for details see [37]). And although tadpoles in the experiments were raised under different water temperatures (colder in Sweden than in Zürich) and at three different food levels, parental genotypes performed worse than hybrid types under all conditions of our study and a previous one by [81]. This lack of a genotype x treatment interaction (cf. Table 4) indicates that at least the environmental conditions tested here cannot be made responsible for the differential offspring survival that leads to the gradual disappearance of LL and RR animals from the population.

### Post-zygotic selection: genetic mechanisms

In terms of genetic post-zygotic selection mechanisms, inviability, reduced fertility and other developmental abnormalities in offspring resulting from hybrid-hybrid matings are generally attributed to an accumulation of deleterious mutations on clonally transmitted genomes through Muller’s ratchet [82]. Such mutational load can act in two not mutually exclusive ways: 1) through homozigosity of recessive deleterious alleles at particular loci or 2) by the cumulative load, and hence general deterioration, of the clonal genomes, independent of homozigosity [26, 36]. Crossing experiments that explicitly addressed these two explanations provided more support for the first mechanism [19, 25, 26]. At a first glance neither mechanism seems to offer an explanation for the gradual disappearance of LL and RR offspring from hybrid x hybrid matings, because reproduction is basically sexual: both the L and the R genome are regularly recombined in triploid individuals, the L when in LLR and the R when in LRR [39, 41, 83]. Therefore, deleterious mutations can regularly be purged and should not accumulate and get fixed. Nevertheless, homozygosity for deleterious mutations may contribute to the mortality of LL and RR progeny, since fixation and low genetic diversity does exist in our Scandinavian all-hybrid populations, at least at microsatellite loci [81, 83–85]. At present, we do not know what mechanism is responsible for that low genetic diversity. Maybe it is due to repeated population bottlenecks and founder effects after glacial periods when water frogs expanded their range from southern refuge areas northwards [86, 87].

### Evolutionary implications

The formation of diploid LR-sperms by hybrid males observed in our study has been described earlier. Some researchers have dispatched them as unimportant due to their larger size and supposed lower swimming speed and fertilisation success compared to haploid sperm [56, 67, 88]. Christiansen [40] on the other hand highlighted the importance of diploid LR sperm for the structure of all-hybrid populations after comparing predictions from a theoretical model with empirical data. She found that LR sperm production in LRR-rich populations was sufficiently high (22 %) to explain the observed proportion of LRR males. Their formation previously had not been understood, because the usual genesis of LRR from LR eggs and R sperm yields only females. Also, the fertilization of LR-eggs with LR-sperm results in symmetrical viable tetraploid offspring (LLRR), which were found in both, natural ponds and our crossing experiment; and in nature at least a few of them survive to adults of both sexes [37]. Since symmetrical polyploids do not encounter the meiotic problems that triploids and other asymmetrical polyploids are facing during gametogeneis, LLRR hybrids can, at least theoretically, have an important evolutionary perspective by opening the possibility for hybrid speciation. Speciation via polyploid hybrids is common in plants [12, 89, 90], but examples from the animal kingdom are scarce, especially when it comes to vertebrates [1, 2, 18, 91, 92]. Unfortunately, we do not know yet whether and how regularly male and female tetraploids produce the diploid LR gametes that would be required for perpetuating a tetraploid line. The only LLRR male for which gamete production is known, produced haploid R sperm and a few diploid cells of unknown genotypic composition [73]. But even if LLRR individuals of both sexes regularly produced LR gametes, Christiansen’s [40] model predicts that a stable tetraploid population would only be established under a more than a twofold increase in survival or reproductive output of both male and female LLRR. This is an unlikely scenario and may explain why, so far, no all-hybrid *Pelophylax* population with a high proportion of tetraploids has been found, despite extensive sampling all over Europe [31]. Yet, theoretically, conditions could be fulfilled under certain ecological conditions, as exemplified by the Iberian minnow *Squalius alburnoides* where the proportion of tetraploids varies markedly between populations living in different habitats [18, 93].

It has been shown, however, that triploid forms producing diploid gametes in one sex and haploid ones in the other sex can act as a stepping stone towards tetraploidization (triploid bridge; [93–95]). They even may establish stable bisexually reproducing all-triploid populations, as [96] have found in Batura toads (*Bufo pseudoraddei baturae*). Thus, under certain genetic and ecological conditions, hybrids can become evolutionary significant units (ESUs) in the sense that some lineages demonstrate *“highly restricted gene flow from other such lineages within the higher organizational level (lineage) of the species”* [15]. Such is also illustrated by the fact that triploid individuals in all-hybrid *Pelophylax esculentus* populations originate from different gamete combinations: in most populations haploid sperm fuses with diploid eggs, whereas in some others diploid sperm fertilizes haploid eggs [73].

## Conclusions

For two reasons, the existence of all-hybrid populations of the hemiclonal frog *Pelophylax esculentus* has long remained a puzzle. First, it was believed that its hybridogenetic mode of reproduction forces *P. esculentus* (the sexual parasite) to always live in sympatry and mate with one of its parental species (the sexual host). This puzzle was solved when it was detected that triploid hybrids can serve as sexual hosts for diploid hybrids and vice versa. Second, analysis of gamete production patterns and mating behavior in diploid and triploid *P. esculentus* revealed, that offspring of both parental species should regularly be produced in all-hybrid populations; yet, they do not exist among adults. In this study, we solved this second puzzle through a field study and a crossing experiment. From the combined results we conclude that the absence of parental genotypes among adults of all-hybrid populations is due to post-zygotic selection against them, rather than to pre-zygotic mechanisms that might prevent their formation in the first place. For this post-zygotic selection, genetic mechanisms seem to be a more likely explanation than ecological factors. At present, we do not know the nature of these mechanisms, but fixation of deleterious mutations and low genetic diversity resulting from repeated population bottlenecks are likely candidates. Given, that all-hybrid populations with diploid and triploid frogs can be perpetuated in the absence of the parental species, *P. esculentus* can be viewed as an evolutionary significant unit that may be on its way towards hybrid speciation via different possible evolutionary trajectories [31].

## Methods

### Sampling

Our analysis of larval and juvenile development under natural conditions is based on repeated sampling in 12 ponds in Skåne (Southern Sweden) at four different stages: eggs, tadpoles, metamorphs and one-year old juveniles. Eggs were collected between June 1 and June 10, 2003; tadpoles were obtained about 6 weeks later (July 16–21) by catching a random sample with a dip net. Metamorphs were caught by hand between August 5 and August 12 and juveniles in spring and summer of the following year. Originally, we had aimed at collecting at least five egg clutches, 25 tadpoles and 20 metamorphs per pond. Unfortunately, these sample sizes could not be achieved in all ponds, partly because of low numbers (perhaps due to high fish abundance) and partly due to problems in finding the existing eggs, tadpoles and metamorphs when the water was muddy and/or vegetation was dense.

For investigating larval development under experimental conditions, we crossed adult frogs from three ponds in Skåne that were chosen based on different population composition, assessed in 2002 and 2003. Pond 001 was LLR-dominated, 089 LRR-dominated and in pond 011 the two triploid genotypes occurred in equal proportions. A genotype was considered dominant when adult frogs of this type constituted more than 50 % of the adult population in both years. While LLR frogs are slightly biased towards males, LRR frogs are heavily sex-biased towards females, making it difficult to encounter LRR males. All frogs were caught between May 15 and May 19, 2004, at night by hand and kept at the field station of the University of Lund at 10 °C prior to the crossing.

### Genotype determination

Since both parts of the study hinge on correct genotype determination, we combined several techniques to obtain accurate results: microsatellite analysis on tissue, flow cytometry on blood, and morphometric indices. For analyzing the genotypes in the egg stage, we raised a subsample of 15 individuals per clutch at Stensoffa, the field station of the University of Lund, under *ad libitum* food conditions until July 22. This was necessary because analyzable amounts of blood and tissue can only be collected once the tadpoles have reached a certain size (~50 days old). Both sets of tadpoles (those raised from the eggs and those sampled from ponds) were staged for their development according to [97] and then killed with a solution of 3-aminobenzoic acid ethyl ester methanesulfonate (MS-222, 5 g/l, Sigma A5040), because it was not possible to obtain enough blood from living animals. Tissue was collected by cutting off part of the tadpole tail. From metamorphs and juveniles we took a toe clip for microsatellite analysis and a blood sample for flow cytometry analysis. Blood was obtained by cutting the web of a hind foot and collecting the emerging drop with a heparinized capillary. All blood samples were stored in a flow rate calibration (FRC) solution [98], and all tissue samples were kept in 70 % ETOH until lab analysis for the genotype determination was done. Additionally, we measured snout-vent length (SVL) and body mass of the metamorphs and juveniles and in adults also the tibia length (TL) and the callus length (CL).

Genome composition of the to-be crossed adults was initially determined based on morphological indices calculated from SVL, TL and CL (for details see [20, 35, 36]) and flow cytometry and later (i.e., after the crossing) confirmed by microsatellite analysis on tissue samples. For this analysis, we used seven polymorphic microsatellite loci: Ca1b5, Ca5, Ca18 [99], Res16 [100], Ca1b6, Re1CAGA10, Ga1a19 [75]. Alleles at these loci are species-specific, i.e., they can unambiguously be assigned to either the L- or the R-genome. Additionally, the loci Ca1b5, Ca1b6, Ga1a19 and Res16 show gene dosage [43] which - in addition to flow cytometry - provided further information about the exact genotype. Details of the protocol for microsatellite analysis are given by [85]. Flow cytometry on blood samples allowed us to distinguish between LR, LLR, LRR and other ploidy levels (e.g., LLRR, LLL and RRR), because L- and R-genomes have different amounts of DNA [64]; the protocol we used is described in [100]. Because the triploid parental types LLL and RRR were rare (0.4 % of the whole sample), we included them for the analysis in the “normal” parental genotypes (LL and RR, respectively).

If flow cytometry and all microsatellite loci showed the same result, the individual was clearly assigned to one genotype. In some cases, however, the results were ambiguous or contradictory, even after reanalysis, e.g., when some microsatellite loci showed a LLR genotype pattern but others a LRR, or when flow cytometry and most microsatellite loci indicated an LLR genotype, but one locus showed only LR, so there was one allele missing. Cases showing repeatedly such contradictory results were categorized as “uncertain” genotypes. They may have been aneuploid (i.e., have missing or additional chromosome fractions) as suggested by [43] or have had the same number of chromosomes as normal diploid and triploid individuals (2n = 26 or 3n = 39), but in an unusual composition of L or R chromosomes, e.g., LR: L = 12 and R = 14, instead of 13 each [76]. Because of these uncertainties such offspring were not considered in most analyses.

### Crossing design

**Fig. 7:**
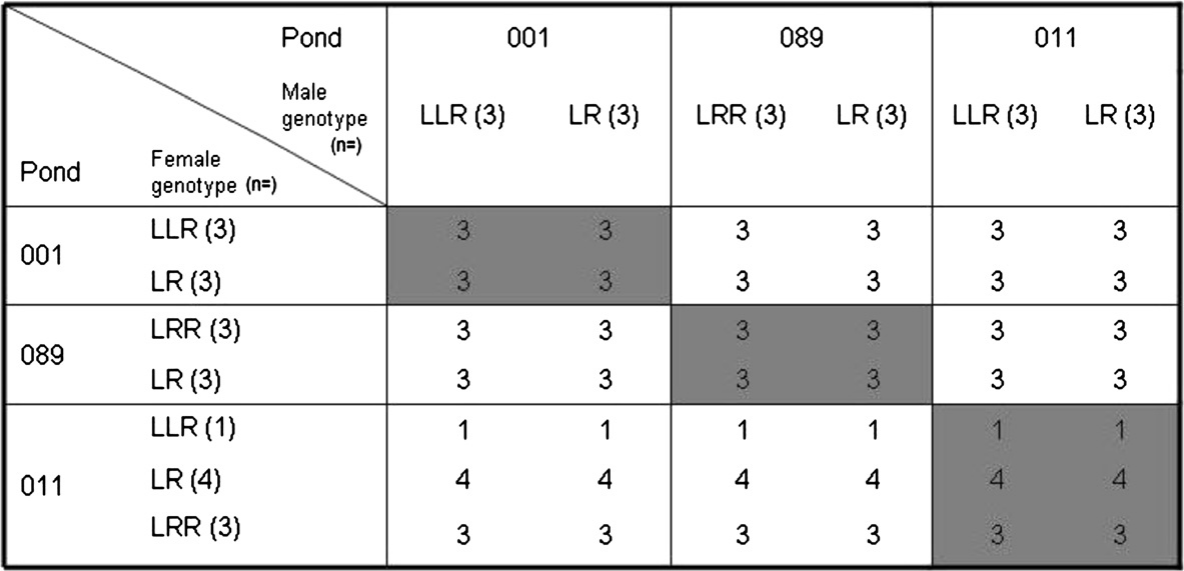
Mating combinations resulting from the experimental crossing design with frogs from three ponds. We included three different individuals per genotype and cross in the experiment with the exception of pond 011, where only one LLR female was caught and one LR individual was initially falsely categorized as a LLR. A total of 120 crossings was carried out. Shaded areas are crosses done within a pond, white areas are crosses done between ponds

Crosses were performed using the artificial fertilization procedure described in detail by [101]. From each of the three ponds we used three males and three females of each available genotype, with the exceptions of pond 011. Here one of the two originally supposed LLR females turned out to be an LR after final genotype analyses, leaving us with only one LLR but four LR females from this pond (Fig. 7). We used only females that were obviously carrying eggs, which was determined according to [102]. Each individual of one sex was crossed with each possible genotype of the other sex within and between ponds. This yielded 120 crossings in the 42 different mating combinations shown in Fig. 7. All crossings were done on the same day. Freshly fertilized eggs were kept in petri dishes filled with filtered pond water. Fertilization success per cross was determined as the proportion of eggs per petri dish that had rotated their black animal hemisphere to the top [101, 103].

On May 23 (1 day after the crossings), the fertilized eggs were transferred from the petri dishes into 1–liter tubs containing aged tap water and then stored in a room at approximately 20 °C. Unfertilized eggs or embryos that stop development can cause degradation of water quality and were therefore removed during regular checking of the tubs. Water was changed twice a week for the same reasons. The larvae were kept indoors until 18 days after fertilization (June 9), when all had reached at least stage 25 which is characterized by the disappearance of external gills [97]. The surviving larvae were weighed, staged and counted, and hatching rate was calculated as the number of hatched larvae relative to the number of fertilized eggs.

### Rearing conditions

On the same day (June 9), we created from each cross four sets of randomly selected tadpoles without any obviously lethal morphological abnormalities and/or feeding and swimming impairments. Set one was used for later genotype determination through microsatellites and flow cytometry as described above. For this analysis, we used 25–56 offspring from each parent individual. The only exception was an LR male from pond 001, for which only two larvae could be analyzed, due to extremely low fertilization success of this male.

Tadpoles of set two were transferred to outdoor tubs at the Stensoffa Fieldstation in Sweden, while those of sets three and four were transported on June 10 by car to the University of Zürich (Switzerland), where on June 11 they were also transferred to outdoor tubs. In both localities, we randomly assigned 15 tadpoles from each cross to tubs of 60 l (Sweden) and 90 l (Zürich), respectively. These tubs had been filled 5–6 weeks earlier with water, provided with a handful of dried leaves and 1–3 snails (*Lymnaea sp*.), and inoculated with phyto- and zooplankton to create a self-sustaining aquatic community [104]. Tadpoles of set two (Sweden) were fed every other day *ad libitum* with rabbit chow (high food treatment); those of set three in Zürich were fed twice a week by adding to each tub 0.5 g rabbit chow (medium food), while those of set four in Zürich could only feed on algae growing naturally in the tub (low food). All tubs were arranged on meadows in a random design and covered with lids to prevent colonization by invertebrate predators.

Eleven percent of the crosses produced not enough viable tadpoles to stock every tub with 15 individuals; in these cases we transferred the available number, ranging from 5 to 14 tadpoles. Five crosses could not be included in further analyses because none of the tadpoles had survived. Larval survival rates under the three food treatments were determined as the proportion of individuals surviving until nine (Sweden) and ten (Zürich) weeks, respectively, after transferring tadpoles to the outdoor tubs. (The 1 week shorter period in Sweden was necessary to avoid time conflicts between raising tadpoles and sampling in natural ponds.) Until then they were counted once a week in Zürich and, on average, every 9 days in Sweden. At the end of this period, we also determined the developmental stage of the surviving animals according to [97]. At this point, the high and low food treatments were terminated and tadpoles preserved for subsequent genotype determination.

Tadpoles from the medium food treatment were returned to their respective tubs and pieces of floating wood added to each tub to allow animals to leave the water after metamorphosis. From then on tubs were checked daily and new metamorphs collected. They were transferred to 1 liter plastic indoor boxes (still separated by cross) with a bottom of wet soil, grass and a few pieces of wood for hiding. In their new environment, animals were kept at room temperature and fed with live crickets twice a week until December 15 when the temperature was gradually lowered to 5 °C over a period of 5 days. After hibernation until early April 2005, temperature was raised to 15 °C within 5 days. All froglets that had survived the winter were transferred to cross-specific 5 liter outdoor boxes, with soil, grass and wooden pieces in one part and water in the other. Here they were kept until July 2005, receiving live crickets twice a week. On June 2, 2005, survival rate and average body mass was determined for each cross, and survival was determined again in July. Thereafter, the experiment was ended by euthanizing the remaining individuals, because due to their origin from artificial crossing between frogs from a foreign country they could not be released into nature.

### Statistical analysis

For our samples from natural populations we calculated the pond-specific proportions of all genotypes for each of the four offspring stages (eggs, tadpoles, metamorphs and juveniles) and tested for differences between them by means of general linear models (PROC GLM), with offspring stage and pond as independent factors. Differences in developmental rates of tadpoles (expressed by Gosner stage, [97]) and in snout-vent length and body mass of metamorphs and juveniles were also analyzed with general linear models.

In our crossing experiment, the produced gamete types were identified by comparing the genotypes of the tadpoles with those of the males and females that were crossed. If an individual produced more than one gamete type, the proportions were calculated as the number of offspring from each gamete type relative to the total number of offspring from this individual.

The early developmental variables (fertilization success and hatching rate) were analyzed with general linear models to test for the effects of female genotype, male genotype, their interaction and crossing type. Genotypes were nested within pond to account for different origins of the same genotypes. Because of missing genotypes, the experiment was not completely balanced and we therefore used Type III sums of squares. The category “crossing type” indicates whether the parents that were mated originated from the same pond (“within ponds”) or from different ponds (“between ponds”).

General linear models were also used to relate larval survival and developmental stage in outdoor tubs, as well as metamorph survival and body mass, to offspring type, food treatment, their interaction and crossing type. For these analyses we used means per cross, including only those crossings where all offspring had the same genotype. For all GLMs we applied post-hoc pairwise Bonferroni tests to investigate which of the genotypes differed. In case of small sample sizes (e.g., few surviving LLRR or LL tadpoles) we pooled offspring types into two categories: hybrids and parental animals. Proportions like genotype ratios in natural ponds and fertilization success, hatchling rate and tadpole and metamorph survival in the experiment were arcsine-square root transformed prior to analyses [105]. All statistical analyses were performed with SYSTAT Version 11 (Systat Software, Inc. 2008).

## Availability of supporting data

The data sets supporting the results of this article are available in the Dryad repository: https://datadryad.org/resource/doi:10.5061/dryad.6rg87.
